# Casting a Wider Net: The Quest for Better Guidance on Seafood Consumption

**DOI:** 10.1289/ehp.120-a244b

**Published:** 2012-06-01

**Authors:** Kellyn S. Betts

**Affiliations:** Kellyn S. Betts has written about environmental contaminants, hazards, and technology for solving environmental problems for publications including *EHP* and *Environmental Science & Technology* for more than a dozen years.

Fish is the primary dietary source of omega-3 long-chain polyunsaturated fatty acids, which are necessary for healthy prenatal development and linked to reducing cardiovascular disease risk. The type and quantity of seafood that individuals choose to eat can impact both their health and the long-term sustainability of the world’s fisheries. An interdisciplinary team of researchers wants consumers to have clearer and more complete guidance in making informed choices about the seafood they eat. Their review presents a framework for producing guidance based not only on nutritional and contaminant information but also ecologic and economic tradeoffs associated with fish consumption choices [*EHP* 120(6):790–798; Oken et al.].

The review highlights areas of overlap and disagreement in current U.S. guidance regarding what constitutes better choices. The myriad fish consumption advice provided by 21 governmental and nongovernmental entities can leave consumers uncertain how to weigh and reconcile multiple benefits and risks associated with any given fish. For instance, the amount of omega-3 fatty acids provided by different fish varies widely, depending on genetics and diet. And where a fish comes from and what it eats can affect its pollutant load—both wild and farmed fish may be high in toxicants such as methylmercury [also see *EHP* 120(6):799–806; Karagas et al.].

**Figure:**
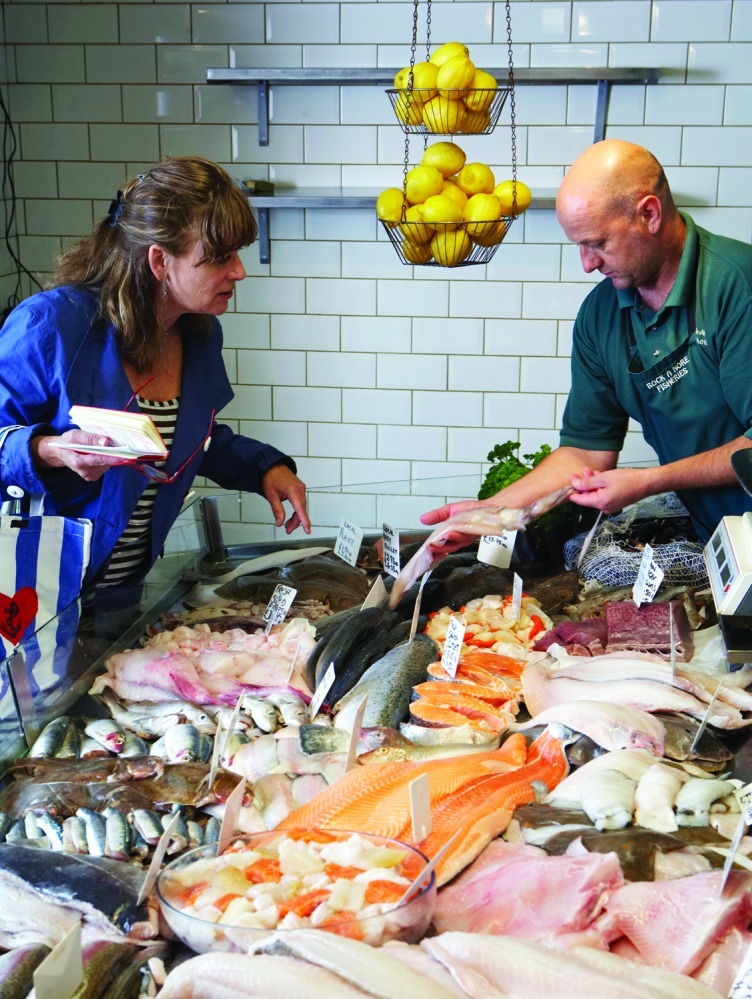
More inclusive fish consumption guides might provide information on ecologic and economic impacts as well as nutritional and contaminant information. © Michael Blann/Thinkstock

The authors posit that fish consumption advice should reflect the reality that both fish farming and the harvesting of wild fish can profoundly affect the health of the oceans. The annual global fish catch exceeds the maximum sustainable yield by three to four times, and some forms of fishing can devastate ocean ecosystems. Aquaculture, the world’s fastest-growing food-production industry, may help ensure adequate fish supplies, but it too has adverse ecological impacts.

The authors recommend that countries develop national lists of fish that can be eaten freely or moderately and fish that should be avoided. These lists should consider all the perspectives analyzed, although the authors acknowledge the challenges of doing so. Ideally, basic national messages would consist of simple lists supplemented by links to more detailed resources for those who desire them, which could be adjusted on a regional basis as necessary. The authors also reaffirm the importance of remediation or elimination of sources of fish contamination as well as policies that promote environmentally responsible and economically viable fishing practices.

